# Suppression of endothelial cell activity by inhibition of TNFα

**DOI:** 10.1186/ar3812

**Published:** 2012-04-25

**Authors:** Qiang Shu, Mohammad A Amin, Jeffrey H Ruth, Phillip L Campbell, Alisa E Koch

**Affiliations:** 1 Department of Internal Medicine, Qilu Hospital of Shandong University, 1107 Jinan Culture Road, Jinan City, China; 2 Department of Internal Medicine, University of Michigan Medical School, 109 Zina Pitcher Drive, Ann Arbor, MI 48109, USA; 3 Department of Internal Medicine, Veterans Administration, 2215 Fuller Road, Ann Arbor, MI 48105, USA

## Abstract

**Introduction:**

TNFα is a proinflammatory cytokine that plays a central role in the pathogenesis of rheumatoid arthritis (RA). We investigated the effects of certolizumab pegol, a TNFα blocker, on endothelial cell function and angiogenesis.

**Methods:**

Human dermal microvascular endothelial cells (HMVECs) were stimulated with TNFα with or without certolizumab pegol. TNFα-induced adhesion molecule expression and angiogenic chemokine secretion were measured by cell surface ELISA and angiogenic chemokine ELISA, respectively. We also examined the effect of certolizumab pegol on TNFα-induced myeloid human promyelocytic leukemia (HL-60) cell adhesion to HMVECs, as well as blood vessels in RA synovial tissue using the Stamper-Woodruff assay. Lastly, we performed HMVEC chemotaxis, and tube formation.

**Results:**

Certolizumab pegol significantly blocked TNFα-induced HMVEC cell surface angiogenic E-selectin, vascular cell adhesion molecule-1 and intercellular adhesion molecule-1 expression and angiogenic chemokine secretion (*P *< 0.05). We found that certolizumab pegol significantly inhibited TNFα-induced HL-60 cell adhesion to HMVECs (*P *< 0.05), and blocked HL-60 cell adhesion to RA synovial tissue vasculature (*P *< 0.05). TNFα also enhanced HMVEC chemotaxis compared with the negative control group (*P *< 0.05) and this chemotactic response was significantly reduced by certolizumab pegol (*P *< 0.05). Certolizumab pegol inhibited TNFα-induced HMVEC tube formation on Matrigel (*P *< 0.05).

**Conclusion:**

Our data support the hypothesis that certolizumab pegol inhibits TNFα-dependent leukocyte adhesion and angiogenesis, probably via inhibition of angiogenic adhesion molecule expression and angiogenic chemokine secretion.

## Introduction

Angiogenesis is a highly regulated process of new blood vessel formation from pre-existing vessels. Angiogenesis is integral to many physiological and pathological processes, but is overactive in disease states such as wound healing, tumor growth [1], cardiovascular disease and rheumatoid arthritis (RA) [2]. The onset of angiogenesis depends on the release of proangiogenic mediators that activate endothelial cells (ECs) and initiate their proliferation and migration [3]. Several types of proangiogenic mediators have been identified to control and balance the initiation and maintenance of angiogenesis. Some of the known angiogenic stimuli include growth factors, such as basic fibroblast growth factor (bFGF) or vascular endothelial growth factor, C-C and C-X-C chemokines [4], and adhesion molecules, such as E-selectin, vascular cell adhesion molecule-1 (VCAM-1) [5], intercellular adhesion molecule-1 (ICAM-1) [6] and junctional adhesion molecules (JAMs). These angiogenic adhesion molecules and chemokines are highly expressed in RA synovial tissues (STs) and synovial fluids [7,8]. Myeloid cells such as monocytes/macrophages circulate in the bloodstream, adhere to ECs, and enter the RA ST, where they release angiogenic mediators, such as TNFα [9].

TNFα is a proinflammatory cytokine implicated in the pathogenesis of a variety of immunological diseases including RA. TNFα appears to orchestrate and perpetuate the inflammatory response in RA, probably by increasing the recruitment of immune cells, mediating the destruction of bone and cartilage [10], and increasing angiogenesis [11]. TNFα upregulates the expression of E-selectin, ICAM-1 [6], VCAM-1 [12], and chemokines, such as monocyte chemoattractant protein-1 (MCP-1)/CCL2 [13], regulated upon activation normal T-cell expressed and secreted (RANTES)/CCL5, growth-related oncogene alpha (Gro-α)/CXCL1 [14], epithelial neutrophil-activating peptide-78 (ENA-78)/CXCL5 [15], granulocyte chemotactic protein-2 (GCP-2)/CXCL6 [16], and IL-8/CXCL8 [14] on ECs. The effect of TNFα on JAMs, including JAM-A, JAM-B and JAM-C, which are enriched at lateral junctions and participate in leucocyte extravasation, especially diapedesis, is still uncertain [17]. Reduction in TNFα improves the signs and symptoms of RA, and the availability of TNFα inhibitors has revolutionized treatment of this illness [18].

Certolizumab pegol is a novel Fc-free, PEGylated, anti-TNFα mAb that binds and neutralizes soluble and transmembrane TNFα [19], and inhibits signaling through both the p55 and p75 TNFα receptors *in vitro*. Certolizumab pegol consists of only the Fab' portion (50 kDa) of a monoclonal antibody directed against TNFα, with humanized framework sequences and a 2 × 20 kDa pegol domain. Certolizumab pegol has demonstrated a fast and lasting effect on the inhibition of joint damage and an improvement of physical function in RA [18]. The ability of certolizumab pegol to mediate cytotoxicity and affect apoptosis of activated human peripheral blood lymphocytes and monocytes has been examined *in vitro *[19], while its effect on angiogenesis is unknown.

We examined the role of TNFα in angiogenesis. We determined that the potential mechanism for the anti-angiogenic activity of certolizumab pegol was in part through blockade of TNFα-induced human dermal microvascular endothelial cell (HMVEC) angiogenic adhesion molecules or chemokines. We also performed cell adhesion assays using human promyelocytic leukemia (HL-60) cells and HMVECs. The effect of certolizumab pegol on HL-60 cell adhesion to RA ST vasculature was evaluated using the Stamper-Woodruff assay [20]. Lastly, HMVEC chemotaxis and tube formation on Matrigel matrix with TNFα were performed. Furthermore, we compared the anti-angiogenic activity using different concentrations of certolizumab pegol. These findings support a role for TNFα modulation of endothelial function, such as leukocyte adhesion and angiogenesis. Our results also show an important novel mechanism for blockade of endothelial function by TNFα inhibitors, such as certolizumab pegol, in RA.

## Materials and methods

### Human dermal microvascular endothelial cells

HMVECs isolated from adult skin capillaries were obtained from Lonza (Walkersville, MD, USA). These cells were cryopreserved at passage 3 by the manufacturer and were routinely cultured for at least 10 population doublings. HMVECs were cultured using complete EC basal medium-2 with EC growth factors (EGM-2 MV Bullet Kit; Lonza). In three series of experiments (cell surface ELISA, cell adhesion, and angiogenic chemokine ELISA) the medium was changed to complete EC basal medium-2 with fetal bovine serum (0.1%) without growth factors 2 hours prior to the experiments.

### Rheumatoid arthritis synovial tissue specimens

STs were obtained from RA patients meeting the American College of Rheumatology criteria [21]. After procurement, the Optimal Cutting Temperature (OCT)-embedded specimens were promptly snap-frozen in liquid nitrogen. Frozen ST samples were cut into ~5 μm sections and stored at -80°C until use. The study was approved by the Institutional Review Board of the University of Michigan Medical School (FWA 00004969). Subjects gave written informed consent prior to participating in the study.

### Cell surface ELISAs for adhesion molecule expression

HMVECs (7.5 × 10^4 ^cells/well) were cultured in 96-well plates (BD Falcon, Bedford, MA, USA) as previously described [22]. Cells were stimulated with TNFα (25 ng/ml; Invitrogen, Grand Island, NY, USA) using several concentrations of certolizumab pegol (UCB, Atlanta, GA, USA) or mouse-IgG (Ms-Ig) as a control (Jackson ImmunoResearch, West Grove, PA, USA), and were incubated for 6 hours for E-selectin and JAM-A and incubated for 24 hours for ICAM-1 and VCAM-1 cell surface expression. Cells were initially fixed with 3.7% formalin in PBS and cell surface ELISAs were performed. Mouse anti-human-E-selectin and anti-human-ICAM-1 antibodies and goat anti-human-VCAM-1 and anti-human-JAM-A antibodies (R&D, Minneapolis, MN, USA) were used at 2.5 μg/ml and plates were read using an ELISA reader (Bio-Rad, Hercules, CA, USA) set at 450 nm. The specificity of the antibodies was confirmed in both the western blot analyses and the ELISAs by the low background and high signal achieved in repeated experiments using both methods and as described by the manufacturer (R&D). The results are shown as the fold change in optical density of stimulated samples to nonstimulated control cells.

### Western blot analysis

HMVECs were stimulated with TNFα (25 ng/ml) in the presence of different concentrations of certolizumab pegol or control Ms-Ig for 24 hours and protein lysates were collected for western blot analysis. Samples were boiled with either reduced or nonreduced loading buffer. Protein concentrations were measured with a BCA protein assay (Thermo Scientific, Rockford, IL, USA). SDS-PAGE was performed with cell lysates after equal protein loading [23]. Antibodies against human E-selectin, ICAM-1 or VCAM-1 (R&D) were incubated overnight in Tris-buffered saline-Tween buffer containing 5% non-fat-milk. Three independent experiments were performed for each adhesion molecule. The results are shown as the fold change of band intensity in treatment samples to nonstimulated control.

### ELISAs for angiogenic chemokines

ECs were incubated with TNFα (25 ng/ml) in the presence or absence of certolizumab pegol or Ms-Ig for 24 hours. HMVEC culture supernatants were collected and ELISAs were performed to determine the concentration of angiogenic chemokines. The chemokines examined were Gro-α/CXCL1, ENA-78/CXCL5, GCP-2/CXCL6, IL-8/CXCL8, MCP-1/CCL2 and RANTES/CCL5. All assays were performed by the University of Michigan Cancer Center Immunology Core, following the manufacturer's protocol (R&D). Samples were run in duplicate for ELISA and were diluted 1:2 to 1:400 in PBS before the assay. PBS served as the negative control.

### Cell adhesion assays *in vitro*

We examined the adhesion of HL-60 cells (American Type Culture Collection, Manassas, VA, USA), a human leukemic myeloid cell line, to HMVECs [24]. ECs were grown in 96-well plates and stimulated with TNFα (25 ng/ml) in the presence of neutralizing antibodies to E-selectin or VCAM-1 or ICAM-1, or with different concentrations of certolizumab pegol or Ms-Ig for 8 hours. Calcein AM (cell-permeant dye, 5 μM; Invitrogen) fluorescent-dye-labeled HL-60 cells (50,000 cells/well in 100 μl RPMI medium) were added to HMVECs and cultures incubated for 30 minutes at 37°C. At the end of the assay, nonadherent cells were washed off, and fluorescence was measured at 485/528 nm using a Synergy HT fluorescence plate reader (BioTek Instruments, Winooski, VT, USA).

### Stamper-Woodruff assay and immunofluorescence staining

Adhesion of HL-60 cells to RA ST vessels was tested as described previously [25]. Briefly, RA STs were incubated with Ms-Ig control (10 μg/ml), certolizumab pegol (10 μg/ml), or anti-E-selectin antibody (10 μg/ml) as a positive control for inhibition of binding to vasculature for 20 hours. Care was taken to select RA STs for each experimental condition with approximately equal amounts of vasculature and size of vessels. This selection ensured that the data evaluated from each group could be appropriately compared, eliminating the possibility that the results may be skewed due to increased vascularity in some tissues. Calcein AM (5 μM)-labeled HL-60 cells (5 × 10^5 ^cells) were then added to each RA section for 30 minutes at room temperature on a rotary agitator (60 rpm). After nonadherent cells were washed off, tissues were fixed with 4% formalin and immunofluorescence staining was performed on RA ST slides with mouse-anti-human von Willebrand factor antibody (500 μg/ml; Dako, Carpinteria, CA, USA), followed by staining with Alexa Fluor 555-conjugated donkey anti-mouse antibody (10 μg/ml; Invitrogen) and nuclei staining with 4',6-diamidino-2-phenylindole (Invitrogen).

Adherent HL-60 cells (green) lying just above the plane of synovial ECs (stained with von Willebrand factor antibody in red) were counted in up to 10 fields, depending on the size and vascularity of the tissue (×200). The adhesion ratio was determined as the sum of adherent HL-60 cells to vessels divided by the sum of blood vessels in up to 10 fields of each section. This was to examine only myeloid cell-vessel interactions to compare the myeloid HL-60 cell binding ratio amongst the different treatment groups, normalized to Ms-Ig. Binding of HL-60 cells to non-ECs was not analyzed. The various treatments are thus presented as the percentage of Ms-Ig binding, defined as the adhesion ratio of the test group divided by the adhesion ratio of the Ms-Ig group.

### HMVEC chemotaxis assays

The HMVEC chemotaxis assays were performed using a modified Boyden chamber to determine whether certolizumab pegol inhibited TNFα-induced EC migration in response to a gradient, a facet of the angiogenic response [26]. HMVECs were preincubated with different concentrations of certolizumab pegol or its Ms-Ig control for 30 minutes before experiments. The stimulus was TNFα at 25 ng/ml with or without corresponding certolizumab pegol or Ms-Ig. bFGF (60 nM; R&D) and PBS served as positive and negative controls, respectively.

### Matrigel tube formation assays

Matrigel is a mixture of extracellular and basement membrane proteins derived from the mouse Engelbreth-Holm-Swarm sarcoma on which ECs attach and rapidly form tubes within 4 to 12 hours. To test the contribution of TNFα in capillary morphogenesis and to examine the role of certolizumab pegol on EC differentiation, we performed EC tube formation assays on growth factor-reduced Matrigel (Becton Dickinson Biosciences, Bedford, MA, USA) in which the levels of stimulatory cytokines and growth factors have been markedly reduced [27]. Four hundred microliters of complete EC basal medium-2 with 0.1% fetal bovine serum containing 16,000 HMVECs (4 × 10^4 ^cells/ml) were added to each well in the presence of different concentrations of TNFα. bFGF (60 nM) and PBS served as positive and negative controls.

An additional series of experiments was performed with TNFα (0.1 ng/ml) using various concentrations of certolizumab pegol or Ms-Ig. After an overnight incubation (18 hours) at 37°C, ECs were fixed and counterstained. Photographs (×40) were taken, and the number of tubes formed was quantitated by an observer blinded to the experimental conditions [27]. Briefly, a connecting branch between two discrete ECs was counted as one tube and required a consistent intensity, thickness, and minimum length (> 2 mm on a ×40 enlarged copy of the photomicrograph) to be counted as a tube.

### Statistical analysis

Data were analyzed using Student's *t *test assuming equal variances. *P *< 0.05 was considered statistically significant. Data are represented as the mean ± standard error of the mean.

## Results

### Certolizumab pegol inhibits TNFα-induced adhesion molecule expression on HMVECs

**Figure 1:**
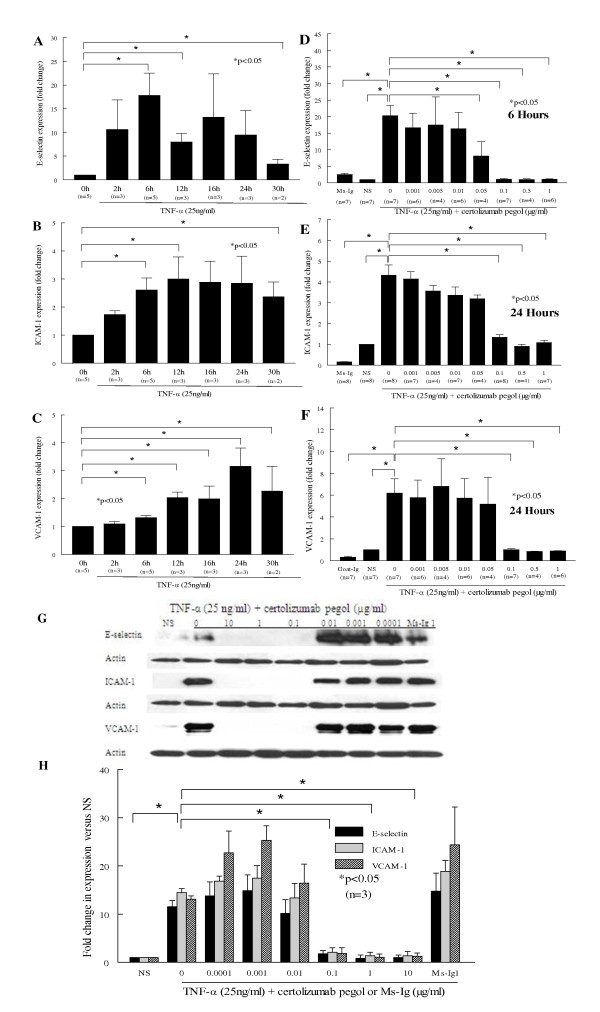
**Certolizumab pegol inhibits TNFα-induced adhesion molecule expression on human dermal microvascular endothelial cells**. E-selectin, intercellular adhesion molecule-1 (ICAM-1) and vascular cell adhesion molecule-1 (VCAM-1) are expressed on human dermal microvascular endothelial cells (HMVECs), inhibited by certolizumab pegol. Cell surface ELISAs were performed to determine the peak expression time of each adhesion molecule on HMVECs induced by TNFα (25 ng/ml). **(A) **E-selectin, **(B) **ICAM-1, and **(C) **VCAM-1 expression is significantly greater on TNFα-stimulated HMVECs compared with the nonstimulated (NS) group. HMVEC expression of E-selectin peaks at 6 hours, while ICAM-1 and VCAM-1 peak at 24 hours (*P < 0.05). Peak expression time points for each adhesion molecule were then used to examine whether certolizumab pegol can inhibit TNFα adhesion molecule induction on HMVECs. **(D) **E-selectin, **(E) **ICAM-1, and **(F) **VCAM-1 expression on HMVECs was stimulated with TNFα (25 ng/ml) in the presence or absence of different concentrations of certolizumab pegol (0.0001 to 10 μg/ml) or mouse-IgG (Ms-Ig) control for 6 hours for E-selectin and for 24 hours for ICAM-1 and VCAM-1 expression, when ECs protein lysates were collected. **(G), (H) **Western blot results indicate that E-selectin, ICAM-1 and VCAM-1 expression are entirely blocked by higher concentrations of certolizumab pegol (0.1 to 10 μg/ml). Blots are representative of three samples. The results are shown as the fold change of treatment samples to the NS group ± standard error of the mean. *n*, number of experiments.

Previous studies have demonstrated that HMVECs have increased expression of select adhesion molecules induced by TNFα [28]. Cell surface ELISAs were performed to determine TNFα (25 ng/ml)-induced endothelial molecules implicated in angiogenesis - namely VCAM-1, ICAM-1, E-selectin, and JAM expression on HMVECs. Our findings indicated that the peak time for E-selectin expression was 6 hours, whereas that for ICAM-1 and VCAM-1 expression on HMVECs was 24 hours (Figure 1A to 1C). We did not find an increase in JAM-A, JAM-B or JAM-C expression on HMVECs when stimulated by TNFα, indicating that not all EC adhesion molecules were TNFα inducible (data not shown). In addition, E-selectin expression at 6 hours, and ICAM-1 and VCAM-1 expression at 24 hours, were all decreased by certolizumab pegol (0.001 to 1 μg/ml) in a concentration-dependent manner (*P *< 0.05) at the maximal time of the respective expression of each of these adhesion molecules (Figure 1D to 1F). These results were confirmed by western blot analyses, which showed that certolizumab pegol (0.1 to 10 μg/ml) completely blocked TNFα-induced adhesion molecule expression on HMVECs (*P *< 0.05; Figure 1G, H).

### Certolizumab pegol inhibits TNFα-induced angiogenic chemokine secretion by HMVECs

**Figure 2:**
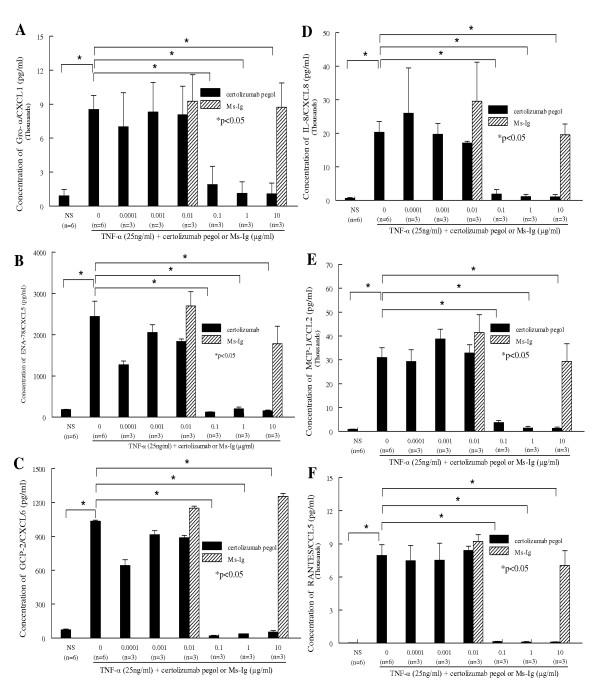
**Certolizumab pegol inhibits TNFα-stimulated human dermal microvascular endothelial cell angiogenic chemokine secretion**. ELISAs were performed to determine the concentrations of angiogenic chemokines in 24-hour TNFα (25 ng/ml)-stimulated culture supernatants. Certolizumab pegol or mouse-IgG (Ms-Ig) control were added to the cultures along with TNFα stimulation. Concentrations of representative samples show that human dermal microvascular endothelial cell (HMVEC) secreted chemokines are significantly upregulated by TNFα (*P < 0.05) and downregulated by certolizumab pegol in a concentration-dependent manner (*P < 0.05). **(A) **Growth-related oncogene alpha (Gro-α)/CXCL1, **(B) **epithelial neutrophil-activating peptide-78 (ENA-78)/CXCL5, **(C) **granulocyte chemotactic protein-2 (GCP-2)/CXCL6, **(D) **IL-8/CXCL8, **(E) **monocyte chemoattractant protein-1 (MCP-1)/CCL2, and **(F) **regulated upon activation normal T-cell expressed and secreted (RANTES)/CCL5. For each set of experiments, means of the concentration of each chemokine are given ± standard error of the mean.

HMVECs were stimulated with TNFα (25 ng/ml) in the presence or absence of different concentrations of certolizumab pegol or Ms-Ig for 24 hours. Cell culture supernatants were collected and a series of ELISAs were performed to determine whether certolizumab pegol inhibits TNFα-induced HMVEC chemokine secretion. We found an increase in concentrations of angiogenic chemokines in the HMVEC supernatants - namely Gro-α/CXCL1, ENA-78/CXCL5, GCP-2/CXCL6, IL-8/CXCL8, MCP-1/CCL2 and RANTES/CCL5 (Figure 2A to 2F). All chemokines listed were increased by TNFα stimulation and inhibited by certolizumab pegol in a dose-dependent manner (*P *< 0.05).

### Certolizumab pegol inhibits HL-60 cell-HMVEC adhesion induced by TNFα

**Figure 3:**
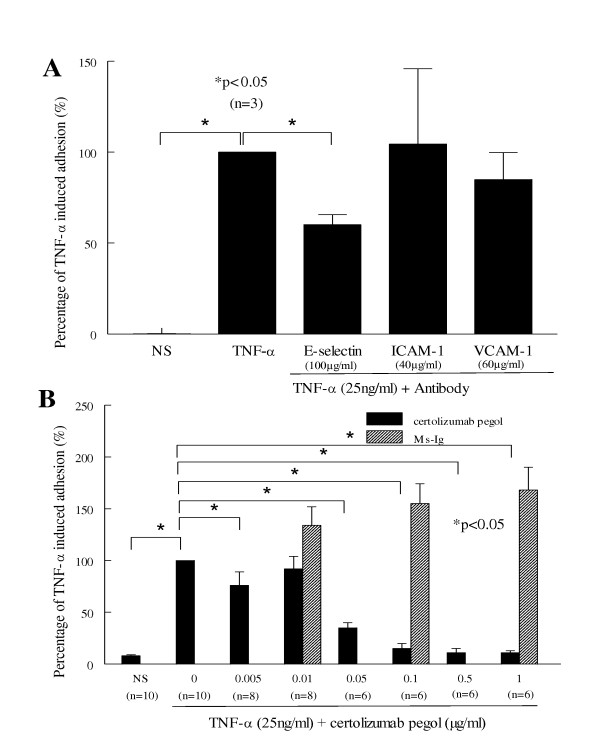
**Certolizumab pegol inhibits HL-60 cell-human dermal microvascular endothelial cell adhesion induced by TNFα**. TNFα-stimulated adhesion-molecule-mediated human promyelocytic leukemia (HL-60) cell-human dermal microvascular endothelial cell (HMVEC) adhesion is blocked by certolizumab pegol. Adhesion assays were performed using HL-60 cells and TNFα-preincubated HMVECs in the presence of anti-E-selectin or anti-intercellular adhesion molecule-1 (anti-ICAM-1) or anti-vascular cell adhesion molecule-1 (anti-VCAM-1) antibody, or of different concentrations of certolizumab pegol or mouse-IgG (Ms-Ig) control for 8 hours. **(A) **TNFα (25 ng/ml) significantly induces HL-60 adhesion to HMVECs, and anti-E-selectin but not anti-ICAM-1 or anti-VCAM-1 antibody block this interaction (*P < 0.05). **(B) **HL-60 cell adhesion to HMVECs is significantly inhibited by certolizumab pegol (0.005 to 1 μg/ml, from 10 to 57% of TNFα-induced binding; *P < 0.05). Results are presented as the percentage of TNFα-induced adhesion ± standard error of the mean. *n*, number of individual experiments. NS, nonstimulated.

We performed HL-60 cell-HMVEC adhesion assays and the result of each treatment group is presented as the percentage of adhering HL-60 cells to TNFα-stimulated ECs. We found that TNFα at 25 ng/ml induced a greater adhesion of myeloid HL-60 cells to ECs than the PBS control group (*P *< 0.05), and this effect was blocked by neutralizing anti-E-selectin but not by anti-VCAM-1 and anti-ICAM-1 antibodies (*P *< 0.05; Figure 3A). This indicates that TNFα-stimulated HL-60 cell-HMVEC adhesion is mediated mainly via E-selectin. Furthermore, our results show that certolizumab pegol (0.005 to 1 μg/ml) decreases TNFα-induced HL-60 cell adhesion (10 to 57% of TNFα-induced binding, *P *< 0.05; Figure 3B).

### Certolizumab pegol inhibits HL-60 cell adhesion to RA synovial tissue vessels

**Figure 4:**
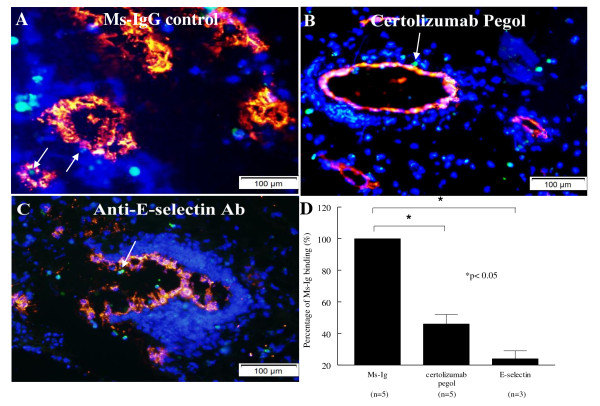
**Certolizumab pegol inhibits HL-60 adhesion to rheumatoid arthritis synovial tissue vessels**. Stamper-Woodruff assay and immunofluorescence were performed using frozen rheumatoid arthritis (RA) synovial tissue (ST) sections and fluorescence-labeled human promyelocytic leukemia (HL-60) cells. Synovial vessels are marked by von Willebrand factor antibody in red; calcein AM-labeled HL-60 cells appear as green dots; and cell nuclei are stained blue with 4',6-diamidino-2-phenylindole. HL-60 cells adhere to the synovial vessels (×200) when treated with: **(A) **mouse-IgG (Ms-Ig; negative control); **(B) **certolizumab pegol (10 μg/ml); or **(C) **anti-E-selectin antibody (Ab; positive control). The arrows in (A) to (C) point to HL-60 cells bound to vasculature. The adhesion ratio of each section was determined as the sum of adherent HL-60 cells divided by the sum of blood vessels in up to 10 fields of each section. **(D) **Adhesion results of the different treatments given as the percentage of Ms-Ig binding, defined as the adhesion ratio of the test group divided by the adhesion ratio of Ms-Ig-treated sections. Results represent the percentage of Ms-Ig binding ± standard error of the mean. *n*, number of RA patients. *P < 0.05.

To determine whether certolizumab pegol plays a functional role in mediating leukocyte adhesion to the RA ST vasculature, we performed *in situ *cell adhesion assays. We found that myeloid HL-60 cells preferentially adhere to blood vessels. The merged photographs of attached HL-60 cells (green) to RA ST vasculature (red) with different treatments are shown for Ms-Ig (negative control; Figure 4A), for certolizumab pegol (10 μg/ml; Figure 4B), or for anti-E-selectin antibody (positive control; Figure 4C).

Note that the arrows in Figure 4A to 4C point to HL-60 cells bound to vasculature. HL-60 cell binding to cells other than ECs was not analyzed. The adhesion seen in the certolizumab pegol group and anti-E-selectin antibody group were 40% and 24% of that seen in the Ms-Ig group, respectively (*P *< 0.05; Figure 4D). Hence, certolizumab pegol inhibited binding of myeloid cells to RA synovial vessels *in situ*, even without exogenous TNFα.

### Certolizumab pegol inhibits TNFα-mediated HMVEC chemotaxis

**Figure 5:**
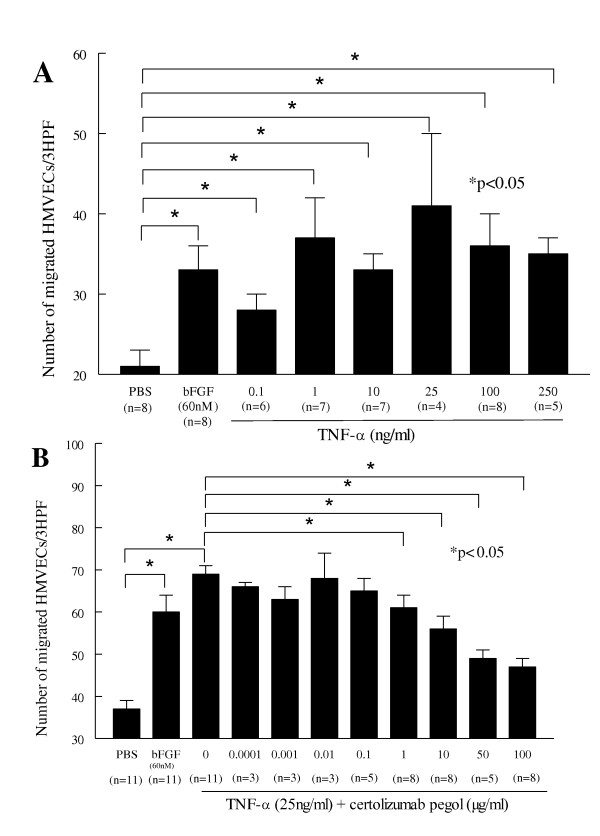
**Certolizumab pegol inhibits TNFα-induced human dermal microvascular endothelial cell chemotaxis**. **(A) **Increasing concentrations of TNFα induce human dermal microvascular endothelial cell (HMVEC) migration, which is significant from concentrations of 0.1 to 250 ng/ml, compared with PBS control (*P < 0.05). **(B) **Certolizumab pegol (1 to 100 μg/ml) decreased TNFα-induced HMVEC migration (from 69% to 89%) when HMVECs were preincubated with different concentrations of certolizumab pegol (*P < 0.05). Cell migration per three high-power fields (3HPF; ×400) was determined by the number of HMVECs migrated towards an angiogenic stimulus. Results represent the mean number of migrating cells per each quadruplicate well ± standard error of the mean. *n*, number of individual experiments. The positive control was basic fibroblast growth factor (bFGF; 60 nM) and PBS was the negative control.

We performed HMVEC chemotaxis assays to test the effect of varying concentrations of TNFα and certolizumab pegol, as HMVEC chemotaxis is one aspect of angiogenesis. Cell migration per three high-power fields (×400) was determined by the number of HMVECs migrated in a modified Boyden chemotaxis chamber towards an angiogenic stimulus. TNFα stimulated HMVEC chemotaxis in a dose-dependent manner, and this migration was significantly greater than migration in the PBS control group (*P *< 0.05; Figure 5A). HMVEC migration in response to TNFα at 25 ng/ml (41 ± 9 cells/well, mean ± standard error of the mean) was comparable with our positive control bFGF group (60 nM; 33 ± 3 cells/well). We preincubated HMVECs with either certolizumab pegol or Ms-Ig at concentrations ranging from 0.0001 to 100 μg/ml for 30 minutes before performing TNFα-induced HMVEC chemotaxis. Certolizumab pegol (1 to 100 μg/ml) resulted in a significant downregulation of HMVEC migration (*P *< 0.05; Figure 5B). Results are representative of three to 11 similar assays.

### Certolizumab pegol inhibits TNFα-induced HMVEC tube formation

**Figure 6:**
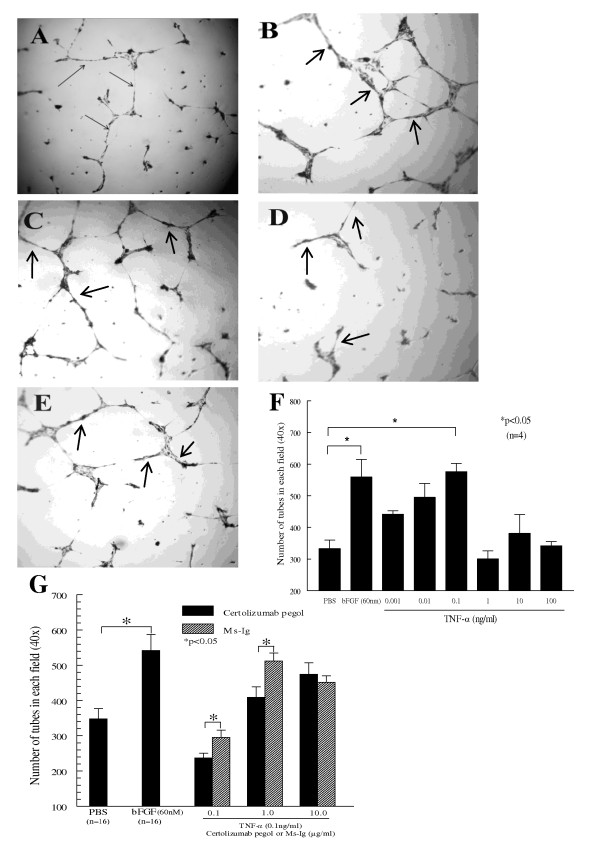
**Certolizumab pegol inhibits TNFα-induced endothelial cell tube formation in Matrigel**. Photomicrographs (×40) of representative wells are shown. The increase in tube formation in the TNFα-induced human dermal microvascular endothelial cell (HMVEC) group and the decreased angiogenesis in certolizumab pegol groups are shown as well as the control groups: **(A) **PBS, **(B) **basic fibroblast growth factor (bFGF; 60 nM), **(C) **TNFα (0.1 ng/ml), **(D) **TNFα (0.1 ng/ml) in the presence of certolizumab pegol (0.1 μg/ml), and **(E) **TNFα (0.1 ng/ml) with mouse-IgG (Ms-Ig; 0.1 μg/ml). Arrows represent the endothelial cell (EC) tube formation in Matrigel. **(F) **HMVECs form significantly greater number of tubes in Matrigel in response to TNFα at 0.1 ng/ml (94% increase) versus the PBS control group (*P < 0.05). **(G) **HMVECs were plated in Matrigel and incubated with either PBS (negative control), bFGF (60 nM; positive control) or TNFα (0.1 ng/ml) with graded concentrations of certolizumab pegol (0.1 μg/ml, *n *= 7; 1.0 μg/ml, *n *= 8; or 10.0 μg/ml, *n *= 4) or, for comparison, Ms-Ig (0.1 μg/ml, *n *= 8; 1.0 μg/ml, *n *= 4; or 10.0 μg/ml, *n *= 4). As shown, certolizumab pegol (0.1 or 1.0 μg/ml) blocks TNFα-induced EC tube formation in Matrigel compared with the Ms-Ig control groups (33% and 30% decreases in tube formation, respectively; *P < 0.05; *n*, number of independent experiments performed). For all sets of experiments, means of the number of tubes per well are given ± standard error of the mean.

When ECs are grown on Matrigel in the presence of an angiogenic substance, this system supports the attachment and differentiation of ECs into tubes in a manner that mirrors the *in vivo *situation [29] and robust EC capillary-like tube formation occurs. To investigate the role of certolizumab pegol and TNFα on EC capillary morphogenesis, we performed EC tube formation on growth factor-reduced Matrigel *in vitro*, in response to TNFα in the presence or absence of certolizumab pegol. PBS (Figure 6A) and bFGF (60 nM; Figure 6B) were used as negative and positive controls, respectively. TNFα (0.1 ng/ml; Figure 6C) induced significant HMVEC tube formation on Matrigel with an increase of 94% compared with the PBS control group (*P *< 0.05). Furthermore, certolizumab pegol at 0.1 μg/ml (Figure 6D) or 0.01 μg/ml blocked TNFα-induced tube formation, causing a 33% and 30% decrease of the TNFα effect, respectively (*P *< 0.05). TNFα (0.1 ng/ml) with Ms-Ig (0.1 μg/ml) also induced EC tube formation, as shown in Figure 6E. Results represent the average of four similar assays at six different concentrations of TNFα, ranging from 0.001 to 100 ng/ml (Figure 6F), and the average of four to 16 similar assays at different concentrations of certolizumab pegol (0.001 and 10 μg/ml, *P *< 0.05; Figure 6G).

## Discussion

Angiogenesis occurs in both physiological and pathological conditions, and plays a key role in synovial inflammation. Adhesion molecules - including selectins, ICAM-1 and VCAM-1, JAMs, and chemokines - regulate vascular permeability and mediate leukocyte adhesion and transmigration, and in some cases angiogenesis [5,6,30]. We and others have shown that these shed adhesion molecules bind adjacent ECs via their respective ligands, exert a direct angiogenic effect on local ECs, and facilitate angiogenesis [5]. E-selectin is highly expressed on endothelium in RA synovium, predominantly on venules and capillaries, whereas VCAM-1 and ICAM-1 are also expressed on other cell types, including ST macrophages, fibroblasts, and lymphocytes in RA synovium compared with osteoarthritis synovium [6,18,31]. TNFα, mainly from monocytes and macrophages [11], promotes inflammation in RA. The concentration of TNFα is elevated in the joints and the blood of RA patients [32]. Animal models support a central role for TNFα in inflammatory arthritis [33]. In many RA patients the clinical benefit of anti-TNFα antibody was prolonged, and appeared to outlast the effective neutralizing level of anti-TNFα in the serum of cA2 (infliximab)-treated individuals. The possible mechanism that may account for these prolonged effects of anti-TNFα could be reduced leucocyte trafficking to the joint [34].

Our results indicate that TNFα upregulates HMVEC E-selectin, VCAM-1, and ICAM-1 expression, measured by cell surface ELISAs and western blot analyses. Certolizumab pegol inhibits HMVEC expression of these adhesion molecules via neutralizing TNFα in a dose-dependent manner. This finding is supported by the fact the TNFα, along with its receptors, can be clearly detected in histology sections, as shown previously in similar RA ST histology sections [32,35], and as such would probably be the driving inducing factor for adhesion molecule expression on the endothelium. Our data are in agreement with previous reports that suggested TNFα increased adhesion molecule expression on ECs [12,36]. Furthermore, we showed that HMVEC expression of these three adhesion molecules is completely inhibited by certolizumab pegol anti-TNFα treatment. Our results agree with the report that infliximab, when given to RA patients, decreased both circulating soluble E-selectin and ICAM-1 concentrations compared with a placebo group [34,37]. Tak and colleagues reported that infliximab decreased the RA ST E-selectin and VCAM-1 expression levels compared with before therapy, which correlated with the degree of disease amelioration in patients [37]. In contrast, soluble VCAM-1 concentrations and RA ST ICAM-1 expression were unaffected by anti-TNFα treatment and were not related to disease activity [37,38]. The difference in results between TNFα blockade with various TNFα blockers may be due to the production of TNFα by other cells, such as macrophages. It may also be due to potential differences in drug bioavailability and the mechanism of action.

JAMs participate in regulating leukocyte transendothelial migration [17]. Some studies reported that TNFα enhanced soluble JAM-A expression on ECs, while we and other authors found TNFα did not increase JAM-A, JAM-B or JAM-C surface expression as assessed by fluorescence-activated cell sorting, cell surface ELISA, and western blot [17,39,40]. TNFα causes the redistribution of JAM-A away from lateral junctions to the cell surface and disperses instead of influencing JAM expression [17,39,40]. Leukocyte trafficking requires not only expression of adhesion molecules by ECs but also a second signal, provided by chemotactic factors such as chemokines. Chemokines anchor to the cell surface, thereby ensuring relatively high concentrations of chemoattractants close to the blood vessel wall, and thus inducing leukocyte infiltration. Furthermore, many of these chemokines - such as Gro-α/CXCL1, ENA-78/CXCL5, GCP-2/CXCL6, IL-8/CXCL8, MCP-1/CCL2 and RANTES/CCL5 - are angiogenic, and are able to induce EC chemotaxis and tube formation [41,42]. MCP-1/CCL2 [13], Gro-α/CXCL1 [16], ENA-78/CXCL5 [15], and IL-8/CXCL8 levels are elevated in RA synovial fluid and serum compared with osteoarthritic synovial fluid and normal peripheral blood levels [15]. Moreover, TNFα increases this chemokine expression [27,43] and anti-TNFα in RA patients results in decreased EC production of chemokines, such as IL-8/CXCL8 and MCP-1/CCL2 [44].

Our data show that TNFα increases angiogenic chemokine secretion in HMVECs and that certolizumab pegol inhibits angiogenic chemokine expression via neutralizing TNFα in a dose-dependent manner. Certolizumab pegol concentrations generally > 0.1 μg/ml block TNFα-induced adhesion molecule and chemokine expression on HMVECs. This suggests that certolizumab pegol at this dose can neutralize both soluble and transmembrane TNFα, and can block TNFα-induced EC effects by inhibiting the TNF receptor pathway. As a result, angiogenic adhesion molecule and chemokine expression on ECs is blocked by certolizumab pegol. Previously, cell surface E-selectin, ICAM-1, and VCAM-1 were shown to mediate T-cell and myeloid-cell binding to ECs [45,46]. We found that certolizumab pegol (0.005 to 1 μg/ml) decreased the TNFα-induced HL-60 cell-HMVEC adhesion. In addition, our data indicate that anti-E-selectin antibody decreased TNFα-induced HL-60 cell-HMVEC adhesion, while anti-ICAM-1 or anti-VCAM-1 antibody failed to block cell adhesion. The reason for different blocking results among anti-E-selectin, anti-ICAM-1 and anti-VCAM-1 antibody is still unclear.

Rheumatoid synovitis is characterized by marked mononuclear infiltration and adhesion molecules, such as E-selectin, which participate in monocyte binding to microvasculature in rheumatoid synovium [47]. Our data show that HL-60 cell adherence to ST vessels *in situ *decreases with certolizumab pegol, indicating that certolizumab pegol inhibits TNFα-induced EC adhesion molecule expression and, subsequently, leukocyte-EC adhesion. We also demonstrate that certolizumab pegol blocks leukocyte adhesion to nonvascular sections of the synovium, such as RA fibroblasts, using both the Stamper-Woodruff assay and HL-60 cell-RA synovial fibroblast adhesion assays (data not shown). We performed HMVEC chemotaxis using TNFα as a stimulus in the presence or absence of certolizumab pegol, and found that TNFα induced EC migration in a concentration-dependent manner. TNFα (25 ng/ml) achieved a peak degree of cell migration, which was higher than bFGF, a potent chemotactic stimulus. Our results confirm the dose range of TNFα to induce EC chemotaxis previously [11], which was 0.5 to 500 ng/ml, and peak stimulation of chemotactic activity was 5 to 50 ng/ml. Moreover, our chemotaxis assays demonstrated a concentration-dependent blocking of TNFα-induced EC migration by certolizumab pegol. These data support the hypothesis that TNFα induces EC chemotaxis and that certolizumab pegol can abrogate this effect.

To investigate the role of certolizumab pegol and TNFα on EC capillary morphogenesis, we performed EC tube formation on growth factor-reduced Matrigel *in vitro*. There are conflicting reports on TNFα induction of tube formation [48-50]. Koolwijk and colleagues found that TNFα (2.5 ng/ml) failed to induce HMVEC tube formation in a fibrin matrix [50], while Zhu and colleagues reported that TNFα (1 ng/ml) induced human umbilical vein endothelial cell tube formation in a BD BioCoat™ angiogenesis system (BD Biosciences, Bedford, MA, USA) [48]. To resolve this issue, we examined the effect of different concentrations of TNFα on HMVEC tube formation and found that TNFα induced an angiogenic effect at 0.1 ng/ml. Our data agree with the results of Leibovich and colleagues, who reported that TNFα at lower concentrations induced a capillary-tube-like structure on collagen gels rather than Matrigel, whereas this effect on tube formation was lost at higher concentrations [11]. Our results on TNFα-induced angiogenesis on Matrigel agree with the reports of Zhu and colleagues [48] and Pan and colleagues [49] that low concentrations of TNFα increase tube formation in the BD BioCoat™ angiogenesis system or on collagen gels. These differing reports may be due to different matrixes and/or methods for the tube formation assay. Of note, we demonstrated that certolizumab pegol blocked the formation of EC tubes on Matrigel.

Interestingly, we also show induction of tube formation in Matrigel with a relatively low dose of TNFα, consistent with previously published results [11]. We were also able to significantly inhibit the proangiogenic effects of TNFα with low doses of certolizumab pegol, which was lost at higher concentrations in the presence of the same concentration of TNFα. Also of note was the finding that increased tube formation correlated with increasing amounts of control Ms-IgG, as well as with certolizumab pegol. This effect was not seen in the other assays (for example, cell surface ELISAs, angiogenic chemokine ELISAs or the adhesion assays). One explanation may be that the matrix environment of the Matrigel may keep large proteins such as antibodies stable and in close proximity to the HMVECs, allowing a nonspecific stimulatory process to occur. For instance, nonspecific secretion of vascular endothelial growth factor by HMVECs might cause an enhancement of tube formation in Matrigel at the higher concentrations of Ms-IgG or certolizumab pegol, as ECs are known to express vascular endothelial growth factor [51]. This is indeed a possible hypothesis because we observed more tube formation with increasing amounts of either Ms-Ig or certolizumab pegol, in the presence of the same amount of TNFα. An alternative explanation may be that the IgG antibodies are binding available EC Fc receptors and activating HMVECs to either form tubes or secrete angiogenic factors, as ECs have been shown to express such receptors [52,53]. This may also explain how nonspecific Ms-IgG could stimulate HMVEC tube formation *in vitro*. However, binding of certolizumab pegol to HMVEC Fc receptors is doubtful because certolizumab pegol lacks an Fc region. Nonetheless, at a consistent concentration of TNFα, certolizumab pegol significantly decreased the angiogenic activity of TNFα compared with the Ms-IgG control antibody at two different concentrations - validating the hypothesis that, at least at relatively lower concentrations, certolizumab pegol appears to be an effective inhibitor of TNFα-induced tube formation in Matrigel.

A notable finding was that the effective inhibition ranges of certolizumab pegol in all the assays were different, which may possibly be due to the different numbers of HMVECs used. For example, in a study of certolizumab pegol action on peripheral blood mononuclear cells *in vitro*, the dose range of certolizumab pegol to neutralize soluble and membrane TNFα expression on different cell lines ranged from 0.01 to 1 μg/ml, while the effective dose range of anti-TNFα on peripheral blood mononuclear cell apoptosis, granulocyte membrane integrity and myeloperoxidase release was higher than 10 μg/ml [19]. Accordingly, in our study the effective blocking dose of certolizumab pegol on HMVEC chemotaxis and tube formation assays was different from the doses needed to be an effective TNFα inhibitor in the adhesion molecule expression, chemokine expression, and HL-60 cell-EC adhesion assays.

It is currently unknown whether similar concentrations of certolizumab pegol would have the same effect on TNFα-activated vasculature in an *in vivo *setting. For example, it would be of interest to investigate whether certolizumab pegol could inhibit vascular formation in the joints of a relevant rodent model of arthritis such as K/BxN serum-induced arthritis, collagen-induced arthritis, or rat adjuvant-induced arthritis. Unfortunately, certolizumab pegol cross-reacts poorly with rodent TNFα, and subsequently the therapeutic was approved for use to treat RA without such studies [54]. Regardless, it is interesting to speculate that disruption of arthritis development by targeting TNFα-induced angiogenesis could be a valid, if not potent, therapeutic strategy. Perhaps future clinical trials with access to synovial biopsies from RA patients treated with certolizumab pegol would shed significant light on this issue.

## Conclusion

In summary, we found that certolizumab pegol inhibited HMVEC expression of angiogenic adhesion molecules and decreased HMVEC angiogenic chemokine secretion, which are two independent pathways to deactivate angiogenesis. At the same time, certolizumab pegol downregulated TNFα-induced myeloid cell adhesion to ECs and blocked leukocyte-EC adhesive interactions in RA ST, suggesting a novel role for certolizumab pegol in blocking monocyte adhesion to inflamed synovial vasculature. Lastly, certolizumab pegol blocked TNFα-induced EC chemotaxis and tube formation *in vitro*. Overall, these findings support the notion that certolizumab pegol, upon neutralizing TNFα, acts as a potent anti-angiogenic agent with the capacity to block EC migration and new blood vessel formation in RA.

## Abbreviations

bFGF: basic fibroblast growth factor; EC: endothelial cell; ELISA: enzyme-linked immunosorbent assay; ENA-78: epithelial neutrophil-activating peptide-78; GCP-2: granulocyte chemotactic protein-2; Gro-α: growth-related oncogene alpha; HL-60: human promyelocytic leukemia; HMVEC: human dermal microvascular endothelial cell; ICAM-1: intercellular adhesion molecule-1; IL: interleukin; JAM: junctional adhesion molecule; mAb: monoclonal antibody; MCP-1: monocyte chemoattractant protein-1; Ms-Ig: mouse-IgG; PBS: phosphate-buffered saline; RA: rheumatoid arthritis; RANTES: regulated upon activation normal T-cell expressed and secreted; ST: synovial tissue; TNF: tumor necrosis factor; VCAM-1: vascular cell adhesion molecule-1.

## Competing interests

The authors declare that they have no competing interests.

## Authors' contributions

QS performed all assays with assistance from JHR, MAA and PLC. JHR and AEK participated in the design of the study, and JHR and QS performed the statistical analysis. AEK conceived of the study, and participated in its design and coordination. JHR, MAA and AEK assisted QS with drafting the manuscript. All authors read and approved the final manuscript.
